# Prevalence of cardiovascular disease and risk factors in a rural district of Beijing, China: a population-based survey of 58,308 residents

**DOI:** 10.1186/1471-2458-12-34

**Published:** 2012-01-16

**Authors:** Liu He, Xun Tang, Yan Song, Na Li, Jingrong Li, Zongxin Zhang, Jianjiang Liu, Liping Yu, Haitao Xu, Jianguo Zhang, Yonghua Hu

**Affiliations:** 1 Department of Epidemiology and Biostatistics, Peking University Health Science Center, Beijing 100191, China; 2 Fangshan District Bureau of Health, Beijing 102488, China; 3 The First Hospital of Fangshan District, Beijing 102400, China; 4 Department of Epidemiology, School of Public Health, University of California, Los Angeles CA90095, USA

## Abstract

**Background:**

Cardiovascular disease (CVD) is the leading cause of global disease burden. Although stroke was thought to be more prevalent than coronary heart disease (CHD) in Chinese, the epidemic pattern might have been changed in some rural areas nowadays. This study was to estimate up-to-date prevalence of CVD and its risk factors in rural communities of Fangshan District, Beijing, China.

**Methods:**

A cross-sectional population survey was carried out by stratified cluster sampling. A total of 58,308 rural residents aged over 40 years were surveyed by face-to-face interview and physical examination during 2008 and 2010. The standardized prevalence was calculated according to adult sample data of China's *5th *Population Census in 2000, and the adjusted prevalence odds ratio (POR) was calculated for the association of CHD/stroke with its cardiovascular risk factors in multivariate logistic regression models.

**Results:**

Age- and sex-standardized prevalence was 5.6% for CHD (5.2% in males and 5.9% in females), higher than the counterpart of 3.7% (4.7% in males and 2.6% in females) for stroke. Compared with previous studies, higher prevalence of 7.7%, 47.2%, 53.3% in males and 8.2%, 44.8%, 60.7% in females for diabetes, hypertension and overweight/obesity were presented accordingly. Moreover, adjusted POR (95% confidence interval) of diabetes, obesity, stage 1 and stage 2 hypertension for CHD as 2.51 (2.29 to 2.75), 1.53 (1.38 to 1.70), 1.13 (1.02 to 1.26) and 1.35 (1.20 to 1.52), and for stroke as 2.24 (1.98 to 2.52), 1.25 (1.09 to 1.44), 1.44 (1.25 to 1.66) and 1.70 (1.46 to 1.98) were shown respectively in the multivariate logistic regression models.

**Conclusions:**

High prevalence of CVD and probably changed epidemic pattern in rural communities of Beijing, together with the prevalent cardiovascular risk factors and population aging, might cause public health challenges in rural Chinese population.

## Background

Mortality and disease burden of cardiovascular disease (CVD) is increasing globally; and with demographic shifts, urbanization and changing lifestyles, the number of people with high blood pressure, diabetes, obesity or dyslipidemia may grow larger, suggesting a further increase in CVD in the future [1]. China is also experiencing an epidemic of CVD during recent decades [2]. Coronary heart disease (CHD) and stroke, the two major CVD types, are becoming the first and second leading cause of death among adults in China nowadays. Compared to Western people, different epidemiologic pattern of CVD has long been observed in Chinese, for example, stroke is thought to be more prevalent than CHD in Chinese population, especially in rural areas [3,4].

However, with China's economic growth, population aging, nutritional transition and urbanization, especially in areas nearby metropolis such as Beijing, the epidemic pattern of CVD and its major risk factors might have been changed [5]. Since few recent population-based studies concerning CHD and stroke prevalence are available for Chinese, a population-based cross-sectional study was conducted in Fangshan District, a rural area of Beijing, China. In addition, prevalence of cardiovascular risk factors in this study was also compared with results from the InterASIA (The international collaborative study of cardiovascular diseases of Asia) [6-8] and the 2007 survey among suburban communities in Beijing [5].

## Methods

### Study population

**Figure 1:**
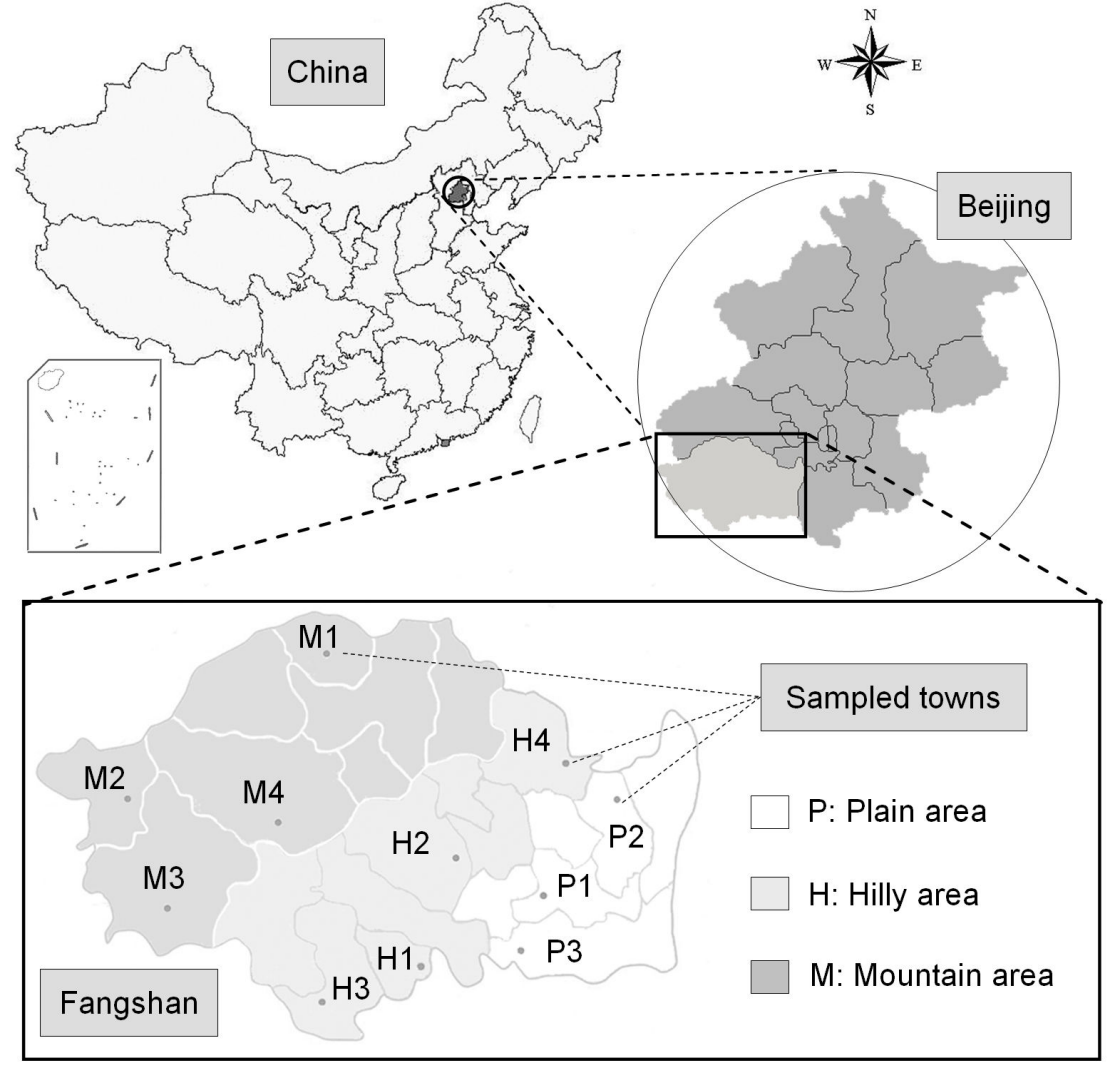
**Geographic map of Fangshan District, Beijing, China**. The spots symbols in the map of Fangshan District indicated the towns selected in this cross-sectional study.

A cross-sectional population survey was carried out from March 2008 through August 2010 in rural communities of Fangshan District, which is located 45 km southwest of downtown Beijing. Briefly, the inclusion criteria for target population in this study are native permanent residents aged over 40 and lived in local communities for at least 5 years. A stratified clustered sampling method was employed. The total 22 regions of Fangshan District were divided into three categories according to geographic characteristics as mountainous area (8 towns), hilly area (8 towns) and plain area (6 towns), and 50% towns were randomly sampled in each part (Figure 1). Thus a total number of 76,544 rural registered inhabitants in the selected towns were enrolled.

This study was approved by the ethics committee of Peking University Health Sciences Center and local institutional review board. Written informed consent was signed by each participant before data collection. Participants with untreated conditions identified during examination were referred to a primary healthcare provider. This study is baseline for the Fangshan/Family-based Ischemic Stroke Study in China (FISSIC) program [9].

### Data collection

Data collection was conducted in the local community healthcare centers in participant's residential area. All participants were interviewed in person by trained investigators to obtain information on demographic characteristics and self-reported medical history including CHD, stroke, diabetes and hypertension using a structured questionnaire (Additional file 1). Then medical records review regarding CHD and stroke were verified by cardiologists and neurologists from the FISSIC program central hospital (the First Hospital of Fangshan District) with patients' records through medical chart review, based on electrocardiography (ECG), computerized tomography (CT) or magnetic resonance imaging (MRI). According to diagnosis standardization from World Health Organization CHD/Stroke Community Registers [10,11], stroke, in this study, was defined as a history of language or physical dysfunction which had been continued for more than 24 h and diagnosed using CT or MRI; and CHD was defined as a history of angina or hospitalization for myocardial infarction with ECG findings, or a surgical history of coronary balloon angioplasty, coronary artery bypass or coronary stent implantation. In addition, medical history of diabetes was defined as self-reported current treatment with insulin or oral hypoglycemic agents.

### Physical examination

During the physical examination, blood pressure was measured at right brachial artery, using standard mercury sphygmomanometers, by qualified physicians or nurses after the participant having been resting in a seated position for at least 5 min before each measurement. Three blood pressure measurements were obtained from each participant according to a common protocol adapted from procedures recommended by the American Heart Association [12], and the average values were used for analysis. Anthropometric measurements were also obtained by trained and certified observers. Height was measured without shoes by a fixed stadiometer and weight without heavy clothing by traditional scales.

### Criteria for data interpretation

Hypertension was defined as systolic blood pressure (SBP) ≥ 140 mmHg, or diastolic blood pressure (DBP) ≥ 90 mmHg, or current use of any antihypertensive medication within 2 weeks or any combination of the above. Blood pressure category was used according to the Seventh Joint National Committee on the Prevention, Detection, Evaluation and Treatment of Hypertension (JNC-7) [13] to classify blood pressure into normotension (SBP < 120 mmHg and DBP < 80 mmHg), prehypertension (SBP of 120-139 mmHg or DBP of 80-89 mmHg), stage 1 hypertension (SBP of 140-159 mmHg or DBP of 90-99 mmHg) and stage 2 hypertension (SBP ≥ 160 mmHg or DBP ≥ 100 mmHg). Body mass index (BMI) was calculated as the ratio of weight to height squared (kg/m^2^). Overweight was defined as BMI ≥ 25 kg/m^2 ^and obesity as BMI ≥ 30 kg/m^2^.

### Statistical analysis

Data analyses were performed using the Statistical Analysis System version 9.1 (SAS Institute Inc., Cary, NC, USA). Continuous measures were expressed as mean ± standard deviation, and other categorical variables were shown as counts and percentages. Prevalence data was directly standardized to the adult sample data of 2000 China *5th *Population Census and 2005 China population for comparison [14]. For each gender, prevalence was standardized using the same age distribution. 95% confidence intervals of prevalence were calculated with PROC SURVEYFREQ from the SAS statistical package. Differences in demographic and anthropometric characteristics, and comparison of age-specific prevalence data between two gender groups were analyzed by *t *test and Chi-square test. The prevalence odds ratio (POR) of cardiovascular risk factors with CHD/stroke was calculated in multivariate logistic regression models, adjusted for age and sex. All tests for statistical significance were two-sided and the significance level was set as *α *= 0.05.

## Results

A total number of 58,308 rural residents (20,362 males and 37,946 females) participated in our study with a response rate of 76.2%. According to the residential geographic classification, 12.0% of participants were living in mountainous areas, 43.5% in hilly areas and 44.5% in plain areas, with mean ages of 58.0 ± 11.2, 55.9 ± 9.9 and 56.2 ± 9.6 years. All participants were classified into 10 years age bands: 40-49 years, 50-59 years, 60-69 years, 70-79 years, 80 years and above, and the numbers (proportion) for each group were 16,767 (28.8%), 21,308 (36.5%), 13,560 (23.3%), 5,755 (9.9%) and 918 (1.6%), respectively. General characteristics of the study participants were shown in Table 1. Compared by sex, the average levels for SBP, DBP, weight and height, and prevalence of hypertension were higher among males, while the counterparts of BMI, diabetes, obesity and overweight were higher in females conversely (all *P *< 0.05). More detailed data was provided in Additional file 2.

**Table 1:** Characteristics of the study participants in Fangshan District, Beijing, China

Characteristics	Total	Males	Females	*P *value*
		
	(*N *= 58,308)	(*N *= 20,362)	(*N *= 37,946)	
Age (years), (mean ± SD)	56.3 ± 9.9	57.1 ± 10.2	55.9 ± 9.8	< 0.001
SBP (mmHg), (mean ± SD)	133.9 ± 17.9	134.4 ± 17.6	133.6 ± 18.0	< 0.001
DBP (mmHg), (mean ± SD)	84.1 ± 10.4	85.1 ± 10.8	83.6 ± 10.2	< 0.001
Weight (kg), (mean ± SD)	67.4 ± 11.4	71.6 ± 11.7	65.3 ± 10.6	< 0.001
Height (cm), (mean ± SD)	160.3 ± 7.9	167.2 ± 6.4	156.6 ± 5.9	< 0.001
BMI (kg/m^2^), (mean ± SD)	26.2 ± 3.8	25.5 ± 3.6	26.6 ± 3.9	< 0.001
BP classification (%), (95% CI)				< 0.001
Normotension	19.3 (16.6, 23.1)	17.8 (15.2, 20.9)	20.7 (17.4, 24.4)	-
Prehypertension	37.8 (36.0, 39.7)	37.7 (35.9, 39.6)	37.9 (36.0, 39.9)	-
Stage 1 hypertension	28.6 (26.3, 30.9)	29.2 (27.1, 31.4)	28.2 (25.8, 30.7)	-
Stage 2 hypertension	13.9 (12.0, 16.1)	15.2 (13.2, 17.5)	13.2 (11.4, 15.3)	-
Hypertension (%), (95% CI)	47.2 (44.5, 49.9)	48.7 (45.6, 51.8)	46.4 (43.8, 49.1)	0.004
Diabetes (%), (95% CI)	8.6 (7.4, 10.0)	7.8 (6.5, 9.3)	9.0 (7.8, 10.4)	< 0.001
Overweight (%), (95% CI)	43.2 (41.7, 44.8)	41.2 (38.9, 43.7)	44.3 (43.1, 45.6)	< 0.001
Obesity (%), (95% CI)	15.1 (13.8, 16.6)	10.7 (9.4, 12.1)	17.5 (16.0, 19.1)	< 0.001

*SBP *systolic blood pressure; *DBP *diastolic blood pressure; *BP *blood pressure; *BMI *body mass index; *SD *standard deviation; *CI *confidence interval. *BP *classification: normotension (SBP < 120 mmHg and DBP < 80 mmHg); prehypertension (SBP of 120-139 mmHg or DBP of 80-89 mmHg); stage 1 hypertension (SBP of 140-159 mmHg or DBP of 90-99 mmHg); stage 2 hypertension (SBP ≥ 160 mmHg or DBP ≥ 100 mmHg)

Hypertension was defined as SBP ≥ 140 mmHg, or DBP ≥ 90 mmHg, or current use of any antihypertensive medication within two weeks or any combination of the above; diabetes was defined as self-reported current treatment with insulin or oral hypoglycemic agents; overweight was defined as BMI ≥ 25 kg/m^2 ^and obesity as BMI ≥ 30 kg/m^2^

*Compared between males and females

**Figure 2:**
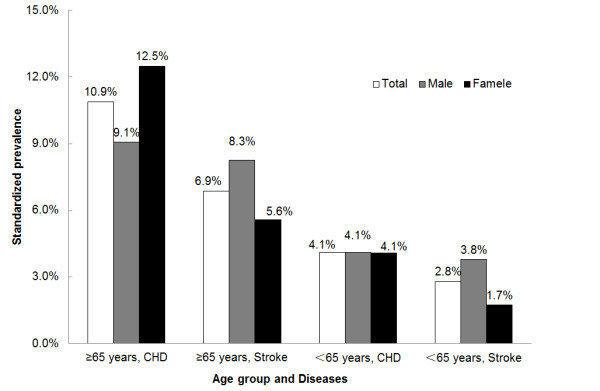
**Standardized prevalence of CHD and stroke among adult population over 65 years and below 65 years in Fangshan District**. Prevalence for all participants was directly standardized to the adult sample data of 2000 China 5th Population Census; Prevalence by genders was age-standardized using the same age distribution from entire adult sample data of 2000 China 5th Population Census.

In participants aged above 40 of this study, overall crude record-verified self-reported prevalence of CHD and stroke was 6.1% (95% CI, 5.5%-6.8%) and 3.7% (95% CI, 3.4%-4.0%), respectively. After directly standardized to data from 2000 China *5th *Population Census, the standardized prevalence of CHD was 5.6% (5.2% in males and 5.9% in females), which was higher than that of stroke as 3.7% (4.7% in males and 2.6% in females) (Table 2). Concerning different age groups, there were similar increased trends of age-specific prevalence with age growing for both CHD and stroke either in men or women. In addition, directly standardized prevalence in participants over 65 years was 10.9% for CHD (9.1% in males and 12.5% in females) and 6.9% for stroke (8.3% in males and 5.6% in females), which was much higher than the counterpart in the group below 65 years (4.1% for CHD and 2.8% for stroke) (Figure 2). With respect to diabetes, hypertension, overweight and obesity, the profile of standardized prevalence and the age-specific prevalence were also displayed in Table 2, while their crude prevalence were 8.6%, 47.2%, 43.2% and 15.1%, respectively. Compared by sex, prevalence of hypertension was higher in males (*P *= 0.04), while that of diabetes, overweight and obesity were higher in females (all *P *< 0.001).

**Table 2:** Age- and sex-specific prevalence of cardiovascular diseases and risk factors among participants (%, and its 95% confidence interval)

Groups	Coronary heart disease	Stroke	Diabetes	Hypertension	Overweight	Obesity
Total^#^	5.6 (4.8, 6.5)	3.7 (3.1, 4.4)	8.0 (6.6, 9.6)	46.1 (42.7, 49.4)	42.8 (40.6, 45.0)	14.1 (12.5, 16.0)
*Males*						
40-49	2.8 (2.5, 3.0)	2.2 (1.9, 2.5)	7.9 (6.9, 9.2)	40.6 (37.5, 43.7)	45.6 (43.2, 47.9)	15.0 (13.5, 16.6)
50-59	5.2 (4.3, 6.1)	5.1 (4.7, 5.4)	8.7 (7.2, 10.6)	47.3 (43.6, 51.0)	43.3 (40.5, 46.1)	11.1 (9.7, 12.7)
60-69	7.7 (6.9, 8.5)	7.7 (6.9, 8.5)	7.7 (6.3, 9.3)	53.8 (51.0, 56.7)	39.6 (36.8, 42.5)	8.1 (6.7, 9.7)
70-79	9.7 (8.2, 11.4)	8.4 (7.3, 9.7)	5.3 (4.1, 6.7)	59.2 (56.0, 62.3)	31.2 (29.2, 33.3)	6.3 (5.1, 7.9)
≥80	9.8 (7.3, 13.0)	7.4 (4.8, 11.2)	5.3 (3.5, 7.8)	59.0 (54.3, 63.5)	24.9 (23.0, 26.8)	5.0 (3.4, 7.3)
Overall*	5.2 (4.4, 6.1)	4.7 (4.1, 5.6)	7.7 (6.3, 9.4)	47.2 (43.6, 50.8)	41.8 (39.1, 44.5)	11.5 (10.0, 13.3)
*Females*						
40-49	2.1 (1.8, 2.5)	0.7 (0.6, 0.9)	4.9 (4.2, 5.6)	33.9 (31.2, 36.8)	45.5 (44.6, 46.5)	16.6 (15.2, 18.2)
50-59	5.2 (4.5, 5.9)	2.4 (2.1, 2.7)	9.5 (8.3, 10.7)	45.4 (42.3, 48.6)	46.6 (45.3, 47.8)	18.9 (17.1, 20.8)
60-69	10.6 (9.9, 11.3)	4.7 (4.0, 5.5)	13.5 (11.4, 15.8)	56.8 (54.0, 59.5)	42.7 (41.2, 44.3)	18.6 (16.8, 20.5)
70-79	12.9 (11.3, 14.8)	5.8 (4.8, 6.9)	10.0 (8.0, 12.5)	63.5 (61.3, 65.6)	37.1 (34.9, 39.4)	13.4 (12.0, 15.1)
≥80	15.0 (12.9, 17.4)	5.9 (3.9, 8.9)	8.3 (6.4, 10.8)	61.7 (56.9, 66.2)	30.0 (25.2, 35.3)	8.3 (5.8, 11.9)
Overall*	5.9 (5.1, 6.9)	2.6 (2.1, 3.1)	8.2 (7.0, 9.7)	44.8 (41.7, 47.9)	43.8 (42.2, 45.4)	16.9 (15.2, 18.8)

Hypertension was defined as SBP ≥ 140 mmHg, or DBP ≥ 90 mmHg, or current use of any antihypertensive medication within 2 weeks or any combination of the above; diabetes was defined as self-reported current treatment with insulin or oral hypoglycemic agents; overweight was defined as BMI ≥ 25 kg/m^2 ^and obesity as BMI ≥ 30 kg/m^2^

^#^Directly standardized to the adult sample data of 2000 China *5th *Population Census

*Both genders were age-standardized using the same age distribution from entire adult sample data of 2000 China *5th *Population Census

**Figure 3:**
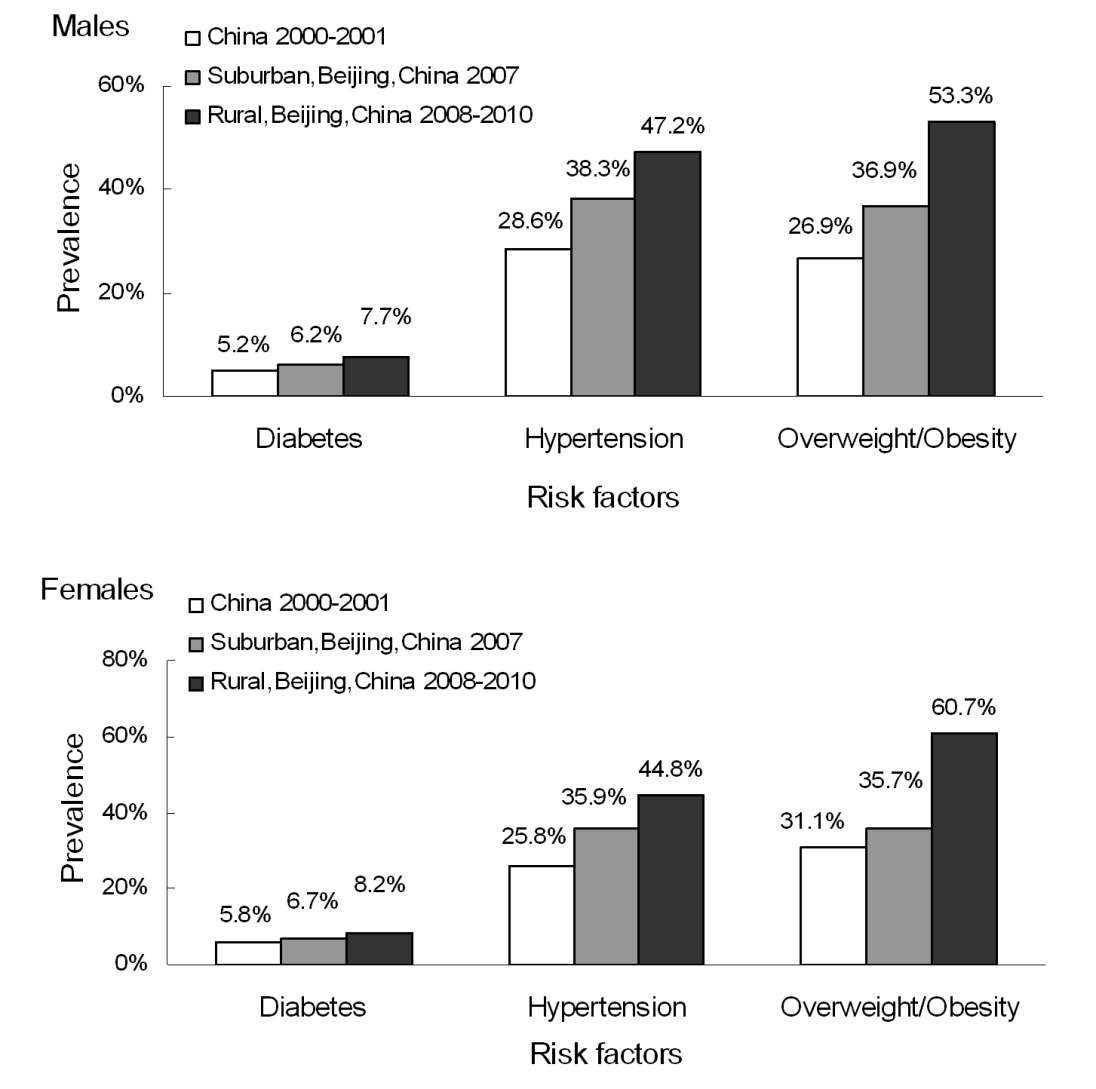
**Comparison of prevalence of diabetes, hypertension and overweight/obesity among males and females in the 2000-2001 InterASIA study, the 2007 survey in suburban of Beijing, and the 2008-2010 survey in rural Beijing**. All the data were directly standardized to the adult sample data of 2000 China *5th *Population Census.

Besides results in our study, a set of previous nationwide data from the 2000-2001 InterASIA study and data from 2007 survey among suburban communities in Beijing, which were also directly standardized to the 2000 China population, were compared in Figure 3. From this comparison, higher prevalence for diabetes, hypertension and overweight/obesity were observed among males and females in both suburban and rural Beijing recently, and even higher in the rural population, especially for overweight/obesity in both sexes. Furthermore, in order to compare with the latest nationwide diabetes prevalence from the 2007-2008 China National Diabetes and Metabolic Disorders Study, the standardized prevalence of diabetes in our study was calculated as 8.0% overall (7.6% in males and 8.3% in females) based on the 2005 China population.

**Figure 4:**
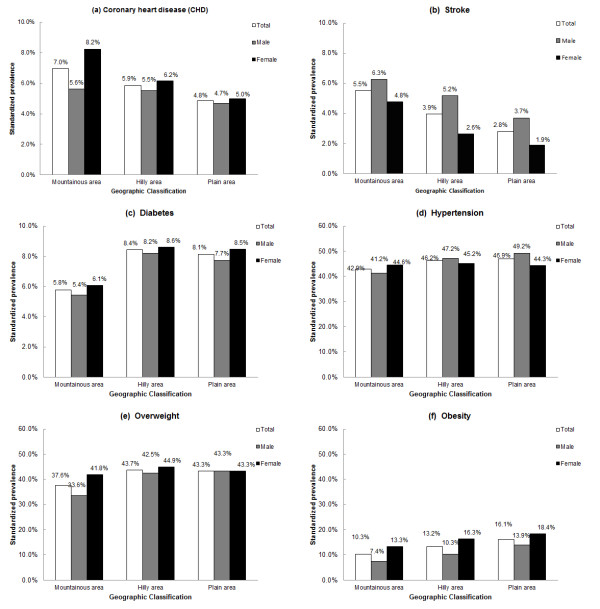
**(**a-f**) Standardized prevalence of CHD (**a**), stroke (**b**), diabetes (**c**), hypertension (**d**), overweight (**e**) and obesity (**f**) by geographic area among adult population older than 40 years in Fangshan District**. Prevalence for all participants was directly standardized to the adult sample data of 2000 China 5th Population Census; Prevalence by genders was age-standardized using the same age distribution from entire adult sample data of 2000 China 5th Population Census.

Among different geographic areas in our study, from mountainous area, hilly area to plain area, with the altitude descending, decreased prevalence of CHD/stroke and increased prevalence of obesity were shown in Figure 4(a-b, f). However, the diversity of CHD prevalence in men was not as obvious as in women. Moreover, compared with mountainous area, higher prevalence of diabetes (Figure 4c), hypertension (Figure 4d) and overweight (Figure 4e) were presented in both hilly area and plain area.

The adjusted PORs of diabetes, hypertension and overweight/obesity with CHD/stroke were shown in Table 3. Statistical analysis rejected the hypothesis of no association between diabetes and CHD (POR = 2.51, 95% CI, 2.29-2.75; *P *< 0.001) or stroke (POR = 2.24, 95% CI, 1.98-2.52; *P *< 0.001). These results indicated significantly that participants with diabetes took more risks of both CHD and stroke. In addition, increased risks for CHD and stroke were also presented in those with overweight/obesity and in persons with hypertension (*P *< 0.01); however, hypertension in each stage indicated higher risk for stroke than for CHD.

**Table 3:** Adjusted associations between coronary heart disease/stroke and cardiovascular risk factors

Variables	Coronary heart disease		Stroke	
	
	Prevalence Odds Ratio (95% CI)	*P *Value	Prevalence Odds Ratio (95% CI)	*P *Value
Diabetes	2.51 (2.29, 2.75)	< 0.001	2.24 (1.98, 2.52)	< 0.001
*BP classification*				
Normotension	Reference	-	Reference	-
Prehypertension	1.04 (0.93, 1.15)	0.505	1.16 (1.01, 1.33)	0.043
Stage 1 hypertension	1.13 (1.02, 1.26)	< 0.001	1.44 (1.25, 1.66)	< 0.001
Stage 2 hypertension	1.35 (1.20, 1.52)	< 0.001	1.70 (1.46, 1.98)	< 0.001
*Weight classification*				
Normal weight	Reference	-	Reference	-
Overweight	1.28 (1.19, 1.39)	< 0.001	1.20 (1.09, 1.33)	< 0.001
Obesity	1.53 (1.38, 1.70)	< 0.001	1.25 (1.09, 1.44)	0.001

*BP *Blood Pressure; *CI *confidence interval. *BP *classification: normotension (SBP < 120 mmHg and DBP < 80 mmHg); prehypertension (SBP of 120-139 mmHg or DBP of 80-89 mmHg); stage 1 hypertension (SBP of 140-159 mmHg or DBP of 90-99 mmHg); stage 2 hypertension (SBP ≥ 160 mmHg or DBP ≥ 100 mmHg)

Diabetes was defined as self-reported current treatment with insulin or oral hypoglycemic agents; overweight was defined as BMI ≥ 25 kg/m^2 ^and obesity as BMI ≥ 30 kg/m^2^

Prevalence odds ratios were calculated with the use of multivariate logistic regression models. All variables listed were included in the model simultaneously, adjusted by age and sex

## Discussion

The present study provided up-to-date prevalence of CVD and its major risk factors among rural adults aged above 40 in Beijing. As one of the strengths, a large community-based stable population was recruited, and the elderly beyond 85 years, who were ignored by most studies, were also included for its highest CVD incidence rate. Furthermore, to enhance the comparability with previous studies, age- and sex-standardized data based on both China population 2000 and 2005 were also provided. In addition, standard protocols and instruments were used, and strict training and a vigorous quality assurance program were conducted to ensure that high-quality data were collected for this study.

The main results of this study demonstrated highly prevalent CHD and stroke in rural areas around a large and fast developing city. Despite lack of data on CHD prevalence, there were several prevalence reports on stroke available for comparison. Previous nationwide studies in China had given a stroke prevalence rate of 0.53% for people aged over 45 years in the 1980s, with higher prevalence in northern cities, such as Beijing, than in the south [15,16]. However, at the very beginning of this century, from National Nutrition and Health Survey, an standardized stroke prevalence of 1.11% (1.26% in male versus 0.96% in female, 1.47% in the north versus 0.72% in the south, and 1.54% in urban versus 0.76% in rural) was reported in general Chinese population aged over 35 years in 2002 [17], which, in spite of being conducted in a relatively younger population, showed an obviously increased stroke prevalence in the past two decades. To add more evidence to the increased trends of CVD, our study provided recent stroke prevalence overall (3.7%), in male (4.7%) and in female (2.6%), which were higher than results of previous studies above, and CHD prevalence (5.6% overall, 5.2% in male and 5.9% in female) in a rural population aged beyond 40 living in North China. CHD and stroke prevalence in China are to some extent indicated to be in trends of rising, and are probably more prominent in northern area. More important, the rural areas around big cities might have been overwhelmed by CVD burden.

Furthermore, a probably changed pattern of CVD prevalence in rural Chinese was presented by the current study. Compared to Western populations, stroke was used to be regarded more common than CHD in Chinese population, especially for rural residents [18,19]. This impression mostly stemmed from the much higher incidence and mortality of stroke than that of CHD based on the Sino-MONICA project conducted from 1987 to 1993, which was one part of WHO MONICA (World Health Organization's Monitoring Trends and Determination in Cardiovascular Disease) project [20]. In the Sino-MONICA project, 7 years surveillance in Beijing indicated incidence and mortality from stroke were 3 times and 1.5 times those of CHD, which would have led to more people with stroke be accumulated than those with CHD, so that higher stroke prevalence would have been observed nowadays. Nevertheless, our study indicated a result of higher CHD prevalence (5.6%) than stroke prevalence (3.7%) recently, which might change the previous knowledge about pattern of CVD in China. Despite lack of comparable data in China and other Asian countries, studies in Japan added evidence to increased incidence and declined mortality for CHD and also declined incidence of stroke in both urban and rural Japanese communities [21,22]. Considering incidence and mortality as two critical factors of prevalence rate, these evidences above suggested a distinctive transition that much heavier burden of CHD might have already existed in Asian countries with a CVD pattern similar to Western countries.

To further describe the CVD epidemic pattern in rural Beijing, data for prevalence of its major risk factors, including diabetes, hypertension and overweight/obesity, were also collected in our study. Hypertension, as an important risk factor of CVD, was found to be highly prevalent among adults in Fangshan District, with much higher prevalence than results from the 2000-2001 InterASIA study [8]. Moreover, a set of increased odds ratios for different blood pressure categories from prehypertension to stage 2 hypertension were also shown to be associated with CHD/stroke, which suggested people with higher blood pressure would be at more risk. In addition, the differences of odds ratios for CHD and stroke indicated a closer relationship between hypertension and stroke, which supported the notion from the INTERSTROKE study published recently [23]. Thus, the prevalent hypertension might contribute to explain relatively high stroke prevalence in rural Chinese. Besides hypertension, diabetes and obesity are also important risk factors of CVD. Although diabetes prevalence in our study was lower than the 2007-2008 China National Diabetes and Metabolic Disorders Study (9.7% in total, 10.6% in males and 8.8% in females) [24], its actual prevalence in Fangshan District might be higher, due to lower awareness of diabetes in rural population in China [25]. The prevalence of overweight and obesity in this study were much higher than national level in 2000-2001 [7]; however, together with other previous evidence [26], this transition reflected the strikingly increase of obesity and might lead to prevalent CHD in China subsequently. Furthermore, prevalence of CVD risk factors in our study was also higher than their counterpart in suburban of Beijing, from a cross-sectional survey in 2007 [5]. Through this comparison, CVD risk factors were found to be more common in rural Beijing, and the remarkable overweight/obesity prevalence probably has a close relationship with nutritional transition in Chinese as previous study indicated [27].

Although it is not the main focus of the study and lack of data from similar researches to compare with, an interesting finding is the diversity in the distribution of CVD and its risk factors across three different geographic areas. Diabetes, hypertension, overweight and obesity prevalence were illustrated to be lower in mountainous area compared with either hilly or plain area, which seemed to be a contradiction with the higher prevalence of CHD and stroke there. It may highlight the importance of other risk factors which were temporarily not explored in our study, such as smoking, family history and lipid profiles. Moreover, it probably also attribute to the inconvenient transportation and low socio-economic status in mountainous area: on the one hand, many labors free of diseases moved from mountainous to plain area, while the sick stayed at home; on the other hand, the poorer medical condition and education level in mountainous area led to the lower awareness of CVD risk factors. Hence lower awareness, treatment and control of CVD risk factors in mountainous area would possibly aggravate the CHD/stroke epidemic.

Besides the major risk factors described above, population aging might be another important force driving the increase of CVD prevalence. It was forecasted that CHD incidences and deaths in China would increase dramatically over 2010-2029, due to population growing and aging alone [28]. Furthermore, the elder population is usually suffering much higher CVD prevalence. Although few data on CHD prevalence for the elders was published so far, there were still a few reliable stroke reports. In some developed countries, the stroke prevalence for people aged 65 years and above ranged from 4.61% to 7.33% (directly standardized to the Segi 1996 world population) [29]. During 2005 national survey in the United States, 8.1% of persons aged 65 years and above reported they had had a history of stroke, whereas the proportion in population aged 45-64 years is 2.7% [30]. In Asia, recent study also pointed out a high prevalence of 10.2% for stroke in residents older than 65 in Korea (directly standardized to the 2005 Korean population) [31]. To make complements, our study provided standardized prevalence of 10.9% for CHD and 6.9% for stroke among participants 65 years and above, much higher than 4.1% for CHD and 2.8% for stroke among individuals below 65 years (directly standardized to the adult sample data of 2000 China *5th *Population Census); and this disparity was even greater in female. Moreover, a higher prevalence of CHD in females aged 65 and above than males was presented in our results, which was inversed from corresponding data of stroke and probably seemed to be contrary to the common ideas; however an earlier population-based study conducted in Hong Kong also reported a much higher CHD prevalence of 9.7% in Chinese women aged 65-74 years than men (5.1%) [32], which was consistent with our study. World population is aging and this has already made a considerable impact on the CVD burden in developed countries in recent decades. It is reasonable to suppose that, as the age structure of the Chinese population increases, the CVD burden in China will be aggravated and might be different by genders.

Urbanization should also be considered as a rational reason to explain higher prevalence of CVD and its risk factors in rural population [24,33]. Though China has the largest population in developing countries with an unbalanced development throughout the whole country, the westernization of lifestyles is accelerated not only among urban residents but also among rural population, especially for the ones nearby modern region [33]. Fangshan District is a typical rural area in the southwest of Beijing, one of the most modernized cities in China, and it might probably be suffered by a high burden of CVD. Thus, taken in this sense, our results may have further public health implications.

Several limitations in our study should be addressed. Firstly, it was a cross-sectional study which could not tell the true relationship between cardiovascular risk factors and CVD. However, it has already been demonstrated in Chinese adults by previous studies [34]. Secondly, twice as many women as men were investigated, probably because part of young and male labor force that went to work in big cities was unavailable for this survey, which is a common problem in rural China. Thirdly, estimation of CHD and stroke prevalence was mainly based on record-verified self-reported data. On the one hand, local general practitioners, who were familiar with health issues of their patients in the serving communities, participated in the investigation to decrease under-reporting level; on the other hand, in order to avoid over-reporting, qualified cardiologists and neurologists were also joined to confirm the diagnosis of diseases through medical record review. Finally, relatively limited information was collected for this large population in the initial baseline, but more detailed information such as surveillance data, life-style risk factors and lipid profiles were scheduled to be added in the next stage as well, so the findings in this study may provide clues for further investigations.

## Conclusions

In summary, this population-based survey indicated a high prevalence of CVD and the probably changed epidemic pattern in Fangshan District, a rural area of Beijing, China. High prevalence of cardiovascular risk factors and population aging might aggravate the burden brought by CVD and become a public health concern in developing rural areas.

## Competing interests

The authors declare that they have no competing interests.

## Authors' contributions

LH, XT and YH conceived of the study, completed all statistical analyses, and drafted the manuscript; YS and NL participated in formulating the study, interpreting the data, and helped to draft the manuscript; JRL, ZZ, JJL LY, HX and JZ carried out the design of the study, collected the data, and helped to revise the manuscript. All authors have read and approved the final manuscript.

## Pre-publication history

The pre-publication history for this paper can be accessed here:

http://www.biomedcentral.com/1471-2458/12/34/prepub

## Supplementary Material

Additional file 1 **Fangshan/Family-based Ischemic Stroke Study in China (FISSIC) program: Questionnaire for baseline survey (English version)**. Click here for file

Additional file 2 **Age- and sex-specific sample size and means ± standard deviations of SBP, DBP, Weight and BMI among participants in Fangshan District, Beijing, China**. Click here for file

## References

[B1] Yusuf S Reddy S Ounpuu S Anand S Global burden of cardiovascular diseases: part I: general considerations, the epidemiologic transition, risk factors, and impact of urbanization Circulation 2001 104 2746 2753 10.1161/hc4601.099487 11723030

[B2] Yang G Kong L Zhao W Wan X Zhai Y Chen LC Koplan JP Emergence of chronic non-communicable diseases in China Lancet 2008 372 1697 1705 10.1016/S0140-6736(08)61366-5 18930526

[B3] Yusuf S Reddy S Ounpuu S Anand S Global burden of cardiovascular diseases: part II: variations in cardiovascular disease by specific ethnic groups and geographic regions and prevention strategies Circulation 2001 104 2855 2864 10.1161/hc4701.099488 11733407

[B4] He J Gu D Wu X Reynolds K Duan X Yao C Wang J Chen CS Chen J Wildman RP Major causes of death among men and women in China N Engl J Med 2005 353 1124 1134 10.1056/NEJMsa050467 16162883

[B5] Zhang L Qin LQ Cui HY Liu AP Wang PY Prevalence of cardiovascular risk factors clustering among suburban residents in Beijing, China Int J Cardiol 2010 151 46 49 20471118 10.1016/j.ijcard.2010.04.056

[B6] Gu D Reynolds K Duan X Xin X Chen J Wu X Mo J Whelton PK He J Prevalence of diabetes and impaired fasting glucose in the Chinese adult population: International collaborative study of cardiovascular disease in Asia (InterASIA) Diabetologia 2003 46 1190 1198 10.1007/s00125-003-1167-8 12879248

[B7] Reynolds K Gu D Whelton PK Wu X Duan X Mo J He J Prevalence and risk factors of overweight and obesity in China Obesity 2007 15 10 18 10.1038/oby.2007.527 17228026

[B8] Gu D Reynolds K Wu X Chen J Duan X Muntner P Huang G Reynolds RF Su S Whelton PK Prevalence, awareness, treatment, and control of hypertension in china Hypertension 2002 40 920 927 10.1161/01.HYP.0000040263.94619.D5 12468580

[B9] Tang X Hu Y Chen D Zhan S Zhang Z Dou H The Fangshan/family-based ischemic stroke study in China (FISSIC) protocol BMC Med Genet 2007 8 60 17825112 10.1186/1471-2350-8-60 PMC1997110

[B10] Tuomilehto J Kuulasmaa K WHO MONICA Project: assessing CHD mortality and morbidity Int J Epidemiol 1989 18 S38 S45 2807706

[B11] Aho K Harmsen P Hatano S Marquardsen J Smirnov VE Strasser T Cerebrovascular disease in the community: results of a WHO collaborative study Bull World Health Organ 1980 58 113 130 6966542 PMC2395897

[B12] Perloff D Grim C Flack J Frohlich ED Hill M McDonald M Morgenstern BZ Human blood pressure determination by sphygmomanometry Circulation 1993 88 2460 2470 8222141 10.1161/01.cir.88.5.2460

[B13] Chobanian AV Bakris GL Black HR Cushman WC Green LA Izzo JL Jr Jones DW Materson BJ Oparil S Wright JT Jr Roccella EJ The seventh report of the joint national committee on prevention, detection, evaluation, and treatment of high blood pressure: the JNC 7 report JAMA 2003 289 2560 2572 10.1001/jama.289.19.2560 12748199

[B14] National Bureau of Statistics of China 1996-2009 Yearly Data of Population http://www.stats.gov.cn/english/statisticaldata/yearlydata/index.htm

[B15] Xue GB Yu BX Wang XZ Wang GQ Wang ZY Stroke in urban and rural areas of China Chin Med J (Engl) 1991 104 697 704 1914638

[B16] Li SC Schoenberg BS Wang CC Cheng XM Bolis CL Wang KJ Cerebrovascular disease in the People's Republic of China: epidemiologic and clinical features Neurology 1985 35 1708 1713 4069361 10.1212/wnl.35.12.1708

[B17] Zhai Y Wang WZ Zhao WH Yang XG Kong LZ The prevalence and onset of age of stroke in Chinese adults Chin J Prev Med 2009 43 1069 1072 in Chinese 20193501

[B18] Zhang XH Lu ZL Liu L Coronary heart disease in China Heart 2008 94 1126 1131 10.1136/hrt.2007.132423 18703693

[B19] Liu M Wu B Wang WZ Lee LM Zhang SH Kong LZ Stroke in China: epidemiology, prevention, and management strategies Lancet Neurol 2007 6 456 464 10.1016/S1474-4422(07)70004-2 17434100

[B20] Wu Z Yao C Zhao D Wu G Wang W Liu J Zeng Z Wu Y Sino-MONICA project: a collaborative study on trends and determinants in cardiovascular diseases in China, part I: morbidity and mortality monitoring Circulation 2001 103 462 468 11157701 10.1161/01.cir.103.3.462

[B21] Iso H Changes in coronary heart disease risk among Japanese Circulation 2008 118 2725 2729 10.1161/CIRCULATIONAHA.107.750117 19106396

[B22] Kitamura A Sato S Kiyama M Imano H Iso H Okada T Ohira T Tanigawa T Yamagishi K Nakamura M Trends in the incidence of coronary heart disease and stroke and their risk factors in Japan, 1964 to 2003: the Akita-Osaka study J Am Coll Cardiol 2008 52 71 79 10.1016/j.jacc.2008.02.075 18582638

[B23] O'Donnell MJ Xavier D Liu L Zhang H Chin SL Rao-Melacini P Rangarajan S Islam S Pais P McQueen MJ Risk factors for ischaemic and intracerebral haemorrhagic stroke in 22 countries (the INTERSTROKE study): a case-control study Lancet 2010 376 112 123 10.1016/S0140-6736(10)60834-3 20561675

[B24] Yang W Lu J Weng J Jia W Ji L Xiao J Shan Z Liu J Tian H Ji Q Prevalence of diabetes among men and women in China N Engl J Med 2010 362 1090 1101 10.1056/NEJMoa0908292 20335585

[B25] Hu D Fu P Xie J Chen CS Yu D Whelton PK He J Gu D Increasing prevalence and low awareness, treatment and control of diabetes mellitus among Chinese adults: the InterASIA study Diabetes Res Clin Pract 2008 81 250 257 10.1016/j.diabres.2008.04.008 18495287

[B26] Wildman RP Gu D Muntner P Wu X Reynolds K Duan X Chen CS Huang G Bazzano LA He J Trends in overweight and obesity in Chinese adults: between 1991 and 1999-2000 Obesity 2008 16 1448 1453 10.1038/oby.2008.208 18388899

[B27] Campbell TC Parpia B Chen J Diet, lifestyle, and the etiology of coronary artery disease: the Cornell China study Am J Cardiol 1998 82 18T 21T 9860369 10.1016/s0002-9149(98)00718-8

[B28] Moran A Zhao D Gu D Coxson P Chen CS Cheng J Liu J He J Goldman L The future impact of population growth and aging on coronary heart disease in China: projections from the coronary heart disease policy model-China BMC Publ Health 2008 8 394 10.1186/1471-2458-8-394 PMC2631484 19036167

[B29] Feigin VL Lawes CM Bennett DA Anderson CS Stroke epidemiology: a review of population-based studies of incidence, prevalence, and case-fatality in the late 20th century Lancet Neurol 2003 2 43 53 10.1016/S1474-4422(03)00266-7 12849300

[B30] The Centers for Disease Control and Prevention Prevalence of stroke----United States, 2005 JAMA 2007 298 279 281

[B31] Han MK Huh Y Lee SB Park JH Lee JJ Choi EA Lim JY Lim S Kim KI Park YJ Prevalence of stroke and transient ischemic attack in Korean elders: findings from the Korean longitudinal study on health and aging (KLoSHA) Stroke 2009 40 966 969 10.1161/STROKEAHA.108.524983 19150874

[B32] Lam TH Liu LJ Janus ED Lau CP Hedley AJ Fibrinogen, angina and coronary heart disease in a Chinese population Atherosclerosis 2000 149 443 449 10.1016/S0021-9150(99)00347-0 10729396

[B33] Wang L Kong L Wu F Bai Y Burton R Preventing chronic diseases in China Lancet 2005 366 1821 1824 10.1016/S0140-6736(05)67344-8 16298221

[B34] Liu J Hong Y D'Agostino RB Sr Wu Z Wang W Sun J Wilson PW Kannel WB Zhao D Predictive value for the Chinese population of the Framingham CHD risk assessment tool compared with the chinese multi-provincial cohort study JAMA 2004 291 2591 2599 10.1001/jama.291.21.2591 15173150

