# Supplementary material for: Prevalence of cardiovascular disease and risk factors in a rural district of Beijing, China: a population-based survey of 58,308 residents
Source: BMC Public Health. 2012 Jan 16;12:34. doi: 10.1186/1471-2458-12-34 (PMC3292979; doi:10.1186/1471-2458-12-34)
Supplement: Additional file 1 — Fangshan/Family-based Ischemic Stroke Study in China (FISSIC) program: Questionnaire for baseline survey (English version). [file 1471-2458-12-34-S1.DOC]

## Age- and sex-specific sample size and means ± standard deviations of SBP, DBP, Weight and BMI among participants in Fangshan District, Beijing, China.

| Age Groups | N | SBP(mmHg) | DBP(mmHg) | Weight(Kg) | BMI(Kg/m^2^) |
| --- | --- | --- | --- | --- | --- |
| Total | 58308 | 133.9 ± 17.9 | 84.1 ± 10.4 | 67.4 ± 11.4 | 26.2 ± 3.8 |
| Male |  |  |  |  |  |
| 40-44 | 2120 | 129.3 ± 16.1 | 86.0 ± 11.5 | 75.9 ± 11.7 | 26.4 ± 3.6 |
| 45-49 | 3329 | 130.3 ± 15.8 | 86.2 ± 10.8 | 74.8 ± 11.4 | 26.2 ± 3.6 |
| 50-54 | 3458 | 132.6 ± 17.1 | 86.1 ± 10.9 | 73.3 ± 11.2 | 25.8 ± 3.5 |
| 55-59 | 3724 | 134.1 ± 17.2 | 85.5 ± 10.5 | 72.0 ± 11.1 | 25.6 ± 3.5 |
| 60-64 | 2982 | 136.8 ± 17.5 | 84.8 ± 10.0 | 70.6 ± 10.9 | 25.3 ± 3.4 |
| 65-69 | 1962 | 138.8 ± 18.2 | 84.3 ± 10.4 | 68.2 ± 11.1 | 24.8 ± 3.5 |
| 70-74 | 1504 | 140.0 ± 19.0 | 83.3 ± 11.1 | 65.6 ± 10.8 | 24.4 ± 3.6 |
| 75-79 | 905 | 139.7 ± 19.0 | 81.8 ± 10.9 | 63.6 ± 10.6 | 24.0 ± 3.5 |
| 80-84 | 308 | 138.7 ± 18.7 | 80.6 ± 9.9 | 62.2 ± 10.8 | 23.7 ± 3.6 |
| ≥85 | 70 | 140.9 ± 20.7 | 82.4 ± 11.3 | 61.2 ± 8.9 | 23.8 ± 3.6 |
| Overall | 20362 | 134.4 ± 17.6 | 85.1 ± 10.8 | 71.6 ± 11.7 | 25.5 ± 3.6 |
| Female |  |  |  |  |  |
| 40-44 | 4610 | 125.7 ± 16.2 | 82.5 ± 10.4 | 66.2 ± 10.2 | 26.3 ± 3.8 |
| 45-49 | 6708 | 129.7 ± 16.9 | 83.9 ± 10.4 | 67.4 ± 10.0 | 26.8 ± 3.7 |
| 50-54 | 6950 | 132.0 ± 17.1 | 84.1 ± 10.0 | 66.7 ± 10.2 | 26.8 ± 3.8 |
| 55-59 | 7176 | 134.5 ± 17.6 | 84.2 ± 9.9 | 66.1 ± 10.5 | 26.9 ± 3.9 |
| 60-64 | 5160 | 137.3 ± 17.8 | 83.9 ± 10.0 | 65.0 ± 10.4 | 26.8 ± 4.0 |
| 65-69 | 3456 | 139.0 ± 17.7 | 82.9 ± 9.8 | 62.5 ± 10.5 | 26.3 ± 4.1 |
| 70-74 | 2126 | 140.7 ± 18.3 | 82.8 ± 10.5 | 60.1 ± 10.7 | 25.8 ± 4.2 |
| 75-79 | 1220 | 142.7 ± 18.7 | 82.7 ± 10.7 | 58.1 ± 10.0 | 25.4 ± 4.0 |
| 80-84 | 419 | 142.9 ± 19.1 | 81.8 ± 10.2 | 55.4 ± 10.6 | 24.7 ± 4.2 |
| ≥85 | 121 | 141.3 ± 19.7 | 80.5 ± 10.0 | 51.0 ± 9.5 | 23.4 ± 3.9 |
| Overall | 37946 | 133.6 ± 18.0 | 83.6 ± 10.2 | 65.3 ± 10.6 | 26.6 ± 3.9 |

SBP, systolic blood pressure; DBP, diastolic blood pressure; BMI, body mass index.
