# Supplementary material for: Prevalence of cardiovascular disease and risk factors in a rural district of Beijing, China: a population-based survey of 58,308 residents
Source: BMC Public Health. 2012 Jan 16;12:34. doi: 10.1186/1471-2458-12-34 (PMC3292979; doi:10.1186/1471-2458-12-34)
Supplement: Additional file 2 — Age- and sex-specific sample size and means ± standard deviations of SBP, DBP, Weight and BMI among participants in Fangshan District, Beijing, China. [file 1471-2458-12-34-S2.DOC]

***Fangshan/Family-based Ischemic Stroke Study In China (FISSIC) program:***

***Questionnaire for baseline survey (English version)***

**Fangshan, Beijing, China**

***Part Ⅰ Basic Information and Disease History***

| 1 | Interviewer ID | **└─┴─┴─┘** |
| --- | --- | --- |

| Location and Date | | Response | | |
| --- | --- | --- | --- | --- |
| 2 | Town ID | **└─┴─┴─┘└─┴─┴─┘** | | |
| 3 | Town name |  | | |
| 4 | Village ID | └─┴─┘ | | |
| 5 | Village name |  | | |
| 6 | Date of completion of the questionnaire | └─┴─┘ └─┴─┘ └─┴─┴─┴─┘  dd mm year | | |
| Consent, Name and Address | | Response | | |
| 7 | Consent has been read and obtained | Yes | 1 |  |
|  |  | No | 2 **If NO, END** |  |
| 8 | Time of interview  (24 hour clock) | └─┴─┘: └─┴─┘  hrs mins | | |
| 9 | Name |  | | |
| 10 | Address |  | | |
| **Additional Information that may be helpful** | | | | |
| 11 | Contact phone number where possible | Home Phone: | | |
|  |  | Mobile Phone: | | |

| **Demographic Information** | | | **Response** | | | |
| --- | --- | --- | --- | --- | --- | --- |
| 12 | | Sex (*Record Male / Female as observed)* | Male | | 1 |  |
|  |  |  | Female | | 2 |  |
| 13 | | What is your date of birth?  *Don't Know 99 99 9999* | └─┴─┘ └─┴─┘ └─┴─┴─┴─┘  dd mm year | | | |
| 14 | | How old are you? | Years **└─┴─┴─┘** | | | |
| **History of Raised Blood Pressure** | | | | | | |
| **Question** | | | **Response** | | | |
| 15 | Have you ever had your blood pressure measured by a doctor or other health worker? | | Yes | 1 | |  |
|  |  |  | No | 2 *If No, go to Question 19* | |  |
| 16 | Have you ever been told by a doctor or other health worker that you have raised blood pressure or hypertension? | | Yes | 1 | |  |
|  |  |  | No | 2  *If No, go to Question 19* | |  |
| 17 | Have you been told that you have raised blood pressure or hypertension in the past 12 months? | | Yes | 1 | |  |
|  |  |  | No | 2 | |  |
| 18 | Are you currently receiving following treatments for high blood pressure prescribed by a doctor or other health worker? | | | | | |
|  | Drugs (medication) that you have taken in the past two weeks | | Yes | 1 | |  |
|  |  |  | No | 2 | |  |
|  | Drug Name 1 | |  | | | |
|  | Drug Name 2 | |  | | | |
| **History of Diabetes** | | | | | | |
| **Question** | | | **Response** | | | |
| 19 | Have you ever had your blood sugar measured by a doctor or other health worker? | | Yes | 1 | |  |
|  |  |  | No | 2 *If No, go to Question 23* | |  |
| 20 | Have you ever been told by a doctor or other health worker that you have raised blood sugar or diabetes? | | Yes | 1 | |  |
|  |  |  | No | 2 *If No, go to Question 23* | |  |
| 21 | Have you been told that you have raised blood sugar or diabetes in the past 12 months? | | Yes | 1 | |  |
|  |  |  | No | 2 | |  |
| 22 | Are you currently receiving any of the following treatments/advice for diabetes prescribed by a doctor or other health worker? | | | | | |
|  | Insulin | | Yes | 1 | |  |
|  |  |  | No | 2 | |  |
|  | Drugs (medication) that you have taken in the past two weeks | | Yes | 1 | |  |
|  |  |  | No | 2 | |  |
|  | Drug Name 1 | |  | | | |
|  | Drug Name 2 | |  | | | |
| **History of Coronary Heart Disease** | | | | | | |
| **Question** | | | **Response** | | | |
| 23 | Have you ever been told by a doctor or other health worker that you have coronary heart disease? | | Yes | 1 | |  |
|  |  |  | No | 2 *If No, go to Question 25* | |  |
|  | Name of the diagnostic hospital | |  | | | |
|  | Time of first diagnosis (year) | |  | | | |
| 24 | Are you currently receiving following treatments for coronary heart disease prescribed by a doctor or other health worker? | | | | | |
|  | Drugs (medication) that you have taken in the past two weeks | | Yes | 1 | |  |
|  |  |  | No | 2 | |  |
|  | Drug Name 1 | |  | | | |
|  | Drug Name 2 | |  | | | |
| **History of Stroke** | | | | | | |
| **Question** | | | **Response** | | | |
| 25 | Have you ever been told by a doctor or other health worker that you have stroke? | | Yes | 1 | |  |
|  |  |  | No | 2 *If No, go to Question 27* | |  |
|  | Name of the diagnostic hospital | |  | | | |
|  | Time of first diagnosis (year) | |  | | | |
| 26 | Are you currently receiving following treatments for stroke prescribed by a doctor or other health worker? | | | | | |
|  | Drugs (medication) that you have taken in the past two weeks | | Yes | 1 | |  |
|  |  |  | No | 2 | |  |
|  | Drug Name 1 | |  | | | |
|  | Drug Name 2 | |  | | | |

***Part Ⅱ Medical Chart Review and Recording for Cardiovascular Disease***

| 27 | Cardiologist ID | **└─┴─┴─┘** |
| --- | --- | --- |
| 28 | Neurologist ID | **└─┴─┴─┘** |

| **Medical Chart Review** | | | | |
| --- | --- | --- | --- | --- |
| **Question** | | **Response** | | |
| 29 | Have the participant ever had any medical record? | Yes | 1 |  |
|  |  | No | 2 *If No, go to Question 40* |  |
| Answer following questions based on **Medical Chart Review**: | | | | |
| 30 | Have the participant ever had any medical record of angina based on electrocardiography (ECG)? | Yes | 1 |  |
|  |  | No | 2 |  |
|  | Have the participant ever had any hospitalization record for myocardial infarction? | Yes | 1 |  |
|  |  | No | 2 |  |
|  | Have the participant ever had any surgical history of coronary balloon angioplasty/coronary artery bypass/coronary stent implantation? | Yes 1  No 2 | |  |
|  | Have the participant ever had any history of language/physical dysfunction which had been continued for more than 24 hours | Yes  No | 1  2 |  |
|  | Have the participant ever had any medical record of stroke based on computerized tomography (CT)? | Yes | 1 |  |
|  |  | No | 2 |  |
|  | Have the participant ever had any medical record of stroke based on magnetic resonance imaging (MRI)? | Yes | 1 |  |
|  |  | No | 2 |  |

| **Medical Chart Recording** | | | | |
| --- | --- | --- | --- | --- |
| 31 | Medical Record Number | |  | |
| 32 | ECG findings: | | | |
| 33 | Date of ECG | | └─┴─┘ └─┴─┘ └─┴─┴─┴─┘  dd mm year | |
| **Paste the ECG Report Here** | | | | |
| 34 | Head CT findings: CT Number **└─┴─┴─┘└─┴─┴─┘** | | | |
| 35 | Date of CT examination | | └─┴─┘ └─┴─┘ └─┴─┴─┴─┘  dd mm year | |
| 36 | Head MRI findings: MRI Number **└─┴─┴─┘└─┴─┴─┘** | | | |
| 37 | Date of MRI examination | | └─┴─┘ └─┴─┘ └─┴─┴─┴─┘  dd mm year | |
| This participant have a confirmed medical history of | | | | |
| 38 | Coronary heart disease | Yes 1  No 2  Not sure 3 | |  |
| 39 | Stroke | Yes 1  No 2  Not sure 3 | |  |

***Part Ⅲ Physical Examination***

| **Height and Weight** | | | | | |
| --- | --- | --- | --- | --- | --- |
| **Question** | | **Response** | | | |
| 40 | Interviewer ID | └─┴─┴─┘ | | | |
| 41 | Device IDs for height and weight | Height | | └─┴─┘ | |
|  |  | Weight | | └─┴─┘ | |
| 42 | Height | in Centimetres (cm) | | └─┴─┴─┘. └─┘ | |
| 43 | Weight  *If too large for scale 666.6* | in Kilograms (kg) | | └─┴─┴─┘.└─┘ | |
| **Blood Pressure** | | | | | |
| **Question** | | **Response** | | | |
| 44 | Interviewer ID |  | └─┴─┴─┘ | | |
| 45 | Device ID for blood pressure |  | └─┴─┘ | | |
| 46 | Cuff size used | Small | 1 | |  |
|  |  | Medium | 2 | |  |
|  |  | Large | 3 | |  |
| 47 | Reading 1 | Systolic ( mmHg) | └─┴─┴─┘ | | |
|  |  | Diastolic (mmHg) | └─┴─┴─┘ | | |
| 48 | Reading 2 | Systolic ( mmHg) | └─┴─┴─┘ | | |
|  |  | Diastolic (mmHg) | └─┴─┴─┘ | | |
| 49 | Reading 3 | Systolic ( mmHg) | └─┴─┴─┘ | | |
|  |  | Diastolic (mmHg) | └─┴─┴─┘ | | |
| 50 | During the past two weeks, have you been treated for raised blood pressure with drugs (medication) prescribed by a doctor or other health worker? | Yes | 1 | |  |
|  |  | No | 2 | |  |
